# Does postoperative quantitative bone scintigraphy reflect outcomes following medial open-wedge high tibial osteotomy?

**DOI:** 10.1371/journal.pone.0257315

**Published:** 2021-09-14

**Authors:** Jung-Kwon Bae, Kang-Il Kim, Jun-Ho Kim, Hyun-Gon Gwak, Chanwoo Kim

**Affiliations:** 1 Department of Orthopaedic Surgery, Kyung Hee University Hospital at Gangdong, Seoul, Korea; 2 Department of Orthopaedic Surgery, Kyung Hee University School of Medicine, Seoul, Korea; 3 Department of Nuclear Medicine, Kyung Hee University Hospital at Gangdong, Seoul, Korea; SickKids Research Institute, CANADA

## Abstract

**Purpose:**

The present study evaluated changes in bone tracer uptake (BTU) after medial open-wedge high tibial osteotomy (MOWHTO) and determined whether postoperative BTU correlates with clinical symptoms, radiologic parameters, or cartilage regeneration following MOWHTO.

**Methods:**

A total of 210 knees underwent MOWHTO for medial compartmental osteoarthritis (OA) were enrolled in this study. Mean follow-up period was 42.7 months. We assessed BTU for the medial compartment of the knee before MOWHTO and at the time of plate removal. Radiologic parameters included Kellgren-Lawrence (K-L) grade and Hip-Knee-Ankle angle (HKAA). Clinical evaluation included American Knee Society (AKS) score and cartilage status was graded at the time of MOWHTO and second-look arthroscopy according to the International Cartilage Repair Society (ICRS) grading system and articular cartilage regeneration stage. Statistical analysis performed to assess the relationships among postoperative BTU of the medial compartment, radiologic parameters, arthroscopic changes and clinical outcomes.

**Results:**

BTU of medial femoral condyle and tibial plateau were significantly decreased at 2 years after MOWHTO (p<0.001). AKS scores and arthroscopic cartilage status were also significantly improved following MOWHTO. BMI and postoperative HKAA showed significant correlations with postoperative changes of BTU in uni- and multi-variable analysis. Meanwhile, postoperative changes of BTU did not show significant correlation with clinical outcomes or cartilage regeneration following MOWHTO.

**Conclusion:**

Lower BMI and postoperative valgus alignment were significant predictor for postoperative BTU decrease of the medial compartment following MOWHTO. However, postoperative changes of BTU did not reflect cartilage regeneration or clinical outcomes until the midterm follow-up.

## 1. Introduction

Medial open-wedge high tibial osteotomy (MOWHTO) is an established treatment for patients with medial compartment knee arthritis combined with varus malalignment [1–4]. Because MOWHTO reduces medial contact pressure by shifting the weight bearing axis from medial to the lateral compartment [5–7], the clinical outcomes after MOWHTO are dependent on the accurate correction of the lower limb alignment according to the preoperative plan [8–10].

Bone scintigraphy has been shown to be useful as part of reflecting knee joint loading pattern [11]. Moreover, bone tracer uptake (BTU) is significantly correlated with mechanical and anatomical alignment of the limb as well as with the degree of osteoarthritis [12]. Thus, it provides great benefits for the assessment of patients after realignment procedure such as MOWHTO [13]. Additionally, bone scintigraphy is sensitive because it reflects the early physiological changes of joints, such as increased blood flow and bone metabolism [14]. Considering the theoretical advantages of bone scintigraphy, our institution has started to use this modality before and after MOWHTO to intuitively identify the effect of MOWHTO in patients with OA, because simple radiographs have limited information to reflect the effect of the operation. Despite of potential and clinical usefulness of bone scintigraphy, there is few reports on correlation with postoperative clinical symptoms, radiologic parameters, or cartilage regeneration following MOWHTO [15].

Therefore, we evaluated changes in BTU following MOWHTO and determined whether postoperative BTU correlates with clinical symptoms, radiologic parameters, or cartilage regeneration. Considering that BTU reflects the loading pattern of the knee joint [11, 12], we hypothesized that medial compartment BTU decreases after MOWHTO, and may correlate with postoperative clinical outcomes and cartilage regeneration.

## 2. Materials and methods

### 2.1 Inclusion and exclusion criteria

The present study was retrospectively collected clinical data from consecutive patients who underwent MOWHTO at our institute from Apr 2013 to Dec 2018. Written informed consent was obtained, and the study protocol was approved by ethics board of Kyung Hee University Hospital at Gangdong (KHNMC 2021-01-048). The inclusion criteria for this study were patients with symptomatic medial compartmental osteoarthritis (OA) with varus malalignment needing to be corrected (more than 5 degrees). Exclusion criteria were as follows: patients (1) had secondary OA such as hemophilic arthropathy, osteonecrosis, joint infection and posttraumatic OA, (2) did not have second-look arthroscopy after MOWHTO, (3) did not have bone scintigraphy prior to initial surgery and at plate removal.

### 2.2 Surgical technique

All patients were operated by a single senior surgeon and received arthroscopic examination at the time of MOWHTO, and the procedure included exploration of the intra-articular structures, removal of loose bodies, and partial meniscectomy for meniscal tears. After arthroscopy, bi-plane osteotomy was carefully performed and the osteotomy gap was determined according to preoperative planning under image intensification [16]. The degree of correction was adjusted by aiming for the mechanical axis near a point at 62.5% of the tibial plateau from the medial edge depending on the arthroscopic cartilage status [17]. We aimed the weight bearing line about 62.5% into the lateral tibial plateau to result in 3°- 4° degrees valgus in advanced arthritis cases. In early arthritic knees, the correction was aimed at shifting the mechanical axis to 50%-60% from the medial edge of plateau [18]. Fixation was carried out through a medial locked plate system (TomoFix^®^; DePuy Synthes, Solothurn, Switzerland). Partial weight-bearing with a crutch typically began on the first postoperative day. Full weight bearing was allowed at postoperative 6 weeks. Almost all patients had same rehabilitation protocol following MOWHTO. Patients were generally recommended to remove the plate with second-look arthroscopy at postoperative 2 years. Arthroscopic procedure was concomitantly performed at the time of plate removal to evaluate the status of knee joint as well as to clean up if necessary.[1, 10, 16, 18–20]

### 2.3 Bone scintigraphy

Bone scintigraphy was conducted before MOWHTO and at the time of the plate removal. The delayed image was obtained 3 to 4 hours after the injection of 925 MBq 99mTc-methylene diphosphonate (MDP) using a dual-head gamma camera (Discovery NM 630, GE, USA) equipped with a low-energy high-resolution collimator. The spot images centered on the knee were subsequently acquired in a 256 x 256 matrix up to 300,000 counts. The tracer count measurement within the ROIs was analyzed by the vendor-provided software (Xeleris 4.0, GE, USA). For reference measurement, a standard region of interest (ROI) with a diameter of 2 cm was positioned centrally in the distal femur 10 cm above the joint space [21]. BTU was calculated by dividing maximal tracer uptake in the ROI of each region by the average tracer uptake in the reference ROI for quantification [15]. Routine analysis of bone scintigraphy were evaluated in the department of nuclear medicine.

### 2.4 Evaluation criteria

Regular follow-up evaluation was performed at the outpatient department at 6 weeks, 3 months, and 1 year after surgery and annually from then on. Bilateral standing anteroposterior and lateral radiographs of the knee and lower extremity scanography were assessed before MOWHTO and every follow-up periods. Radiologic evaluation included Kellgren-Lawrence grade [22], Hip-Knee-Ankle angle (HKAA, ≥ 180° was defined as valgus deformity) [23], correction angle [24], and posterior tibial slope angle (PTSA) [25, 26]. All measurements were taken using a picture archiving and communication system (PiViewSTAR, INFINITT Co, Seoul, Korea). For arthroscopic evaluation, medial femoral condyle and medial tibial plateau were evaluated at the time of MOWHTO and second-look arthroscopy according to the International Cartilage Repair Society (ICRS) grading system [27] and articular cartilage regeneration was classified by the macroscopic staging system because cartilage regeneration has been identified after MOWHTO based on previous studies. [16, 19, 20]. The American Knee Society (AKS) score [28] was used for clinical evaluation. To assess the relative factors influencing postoperative BTU of medial compartment, we analyzed the relationship between postoperative BTU and perioperative variables (sex, age, body mass index (BMI), radiographic values and clinical score).

### 2.5 Statistical analysis

The SPSS software version 23 (IBM Corp, Armonk, NY) was used for statistical analysis with a significance threshold of P < 0.05. All dependent variables were tested for normality of distribution and equality of variance using the Kolmogorov-Smirnov test. The paired t-test was used to analyze normal-distributions. Categorical data were analyzed using the chi-square or Fisher’s exact test. Univariable and multivariable regression analyses were performed to identify factors that influenced postoperative BTU. One-way ANOVA was used to assess the correlation between postoperative BTU and cartilage regeneration. A post-hoc power analysis using G*Power 3.1.9 was used to calculate the overall statistical power of the present study [29]. A post hoc power calculation was also conducted to estimate the minimum sample size required to achieve a power of 80% at 5% alpha level [29]. The post hoc power analysis showed that our tests were overpowered achieved greater than 90% power.; the effect size for BTU of medial femoral condyle and medial tibial plateau was 0.34 and 0.27, respectively, achieving a power of 0.99 for both sides based on 210 cases at 5% alpha level.

## 3. Results

**Fig 1:**
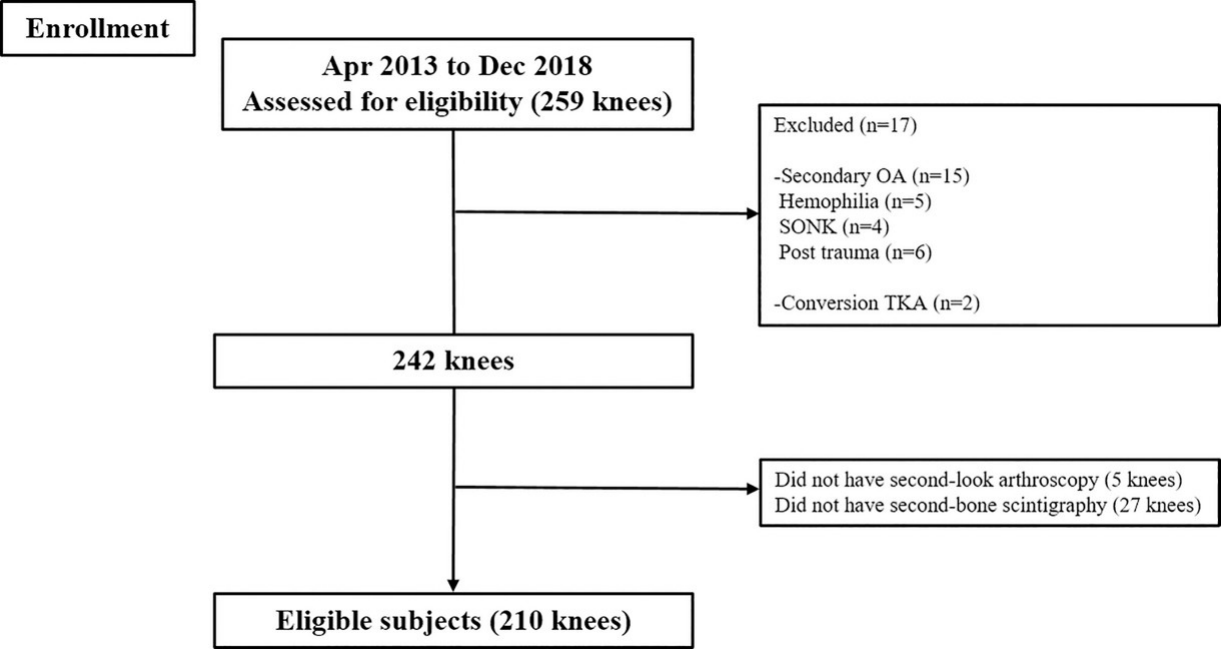
Flow diagram showing the number of knees that met the study criteria.

**Fig 2:**
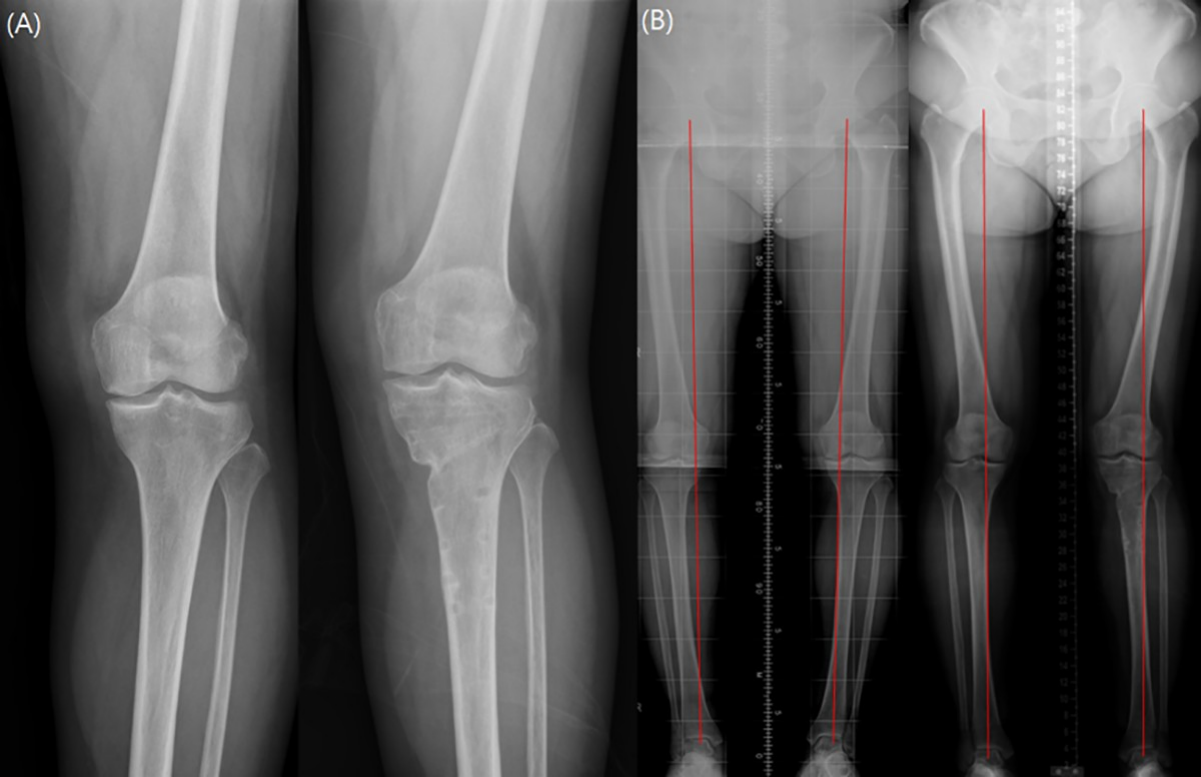
(A) Preoperative (left) and postoperative 2 years (right) standing anteroposterior radiographs of left knee in a 65-year-old female with patient. (B) Preoperative (left) lower extremity orthogram showed 6° varus of left lower limb and postoperative 2 years (right) radiograph showed 3.5° valgus of left lower limb alignment.

**Fig 3:**
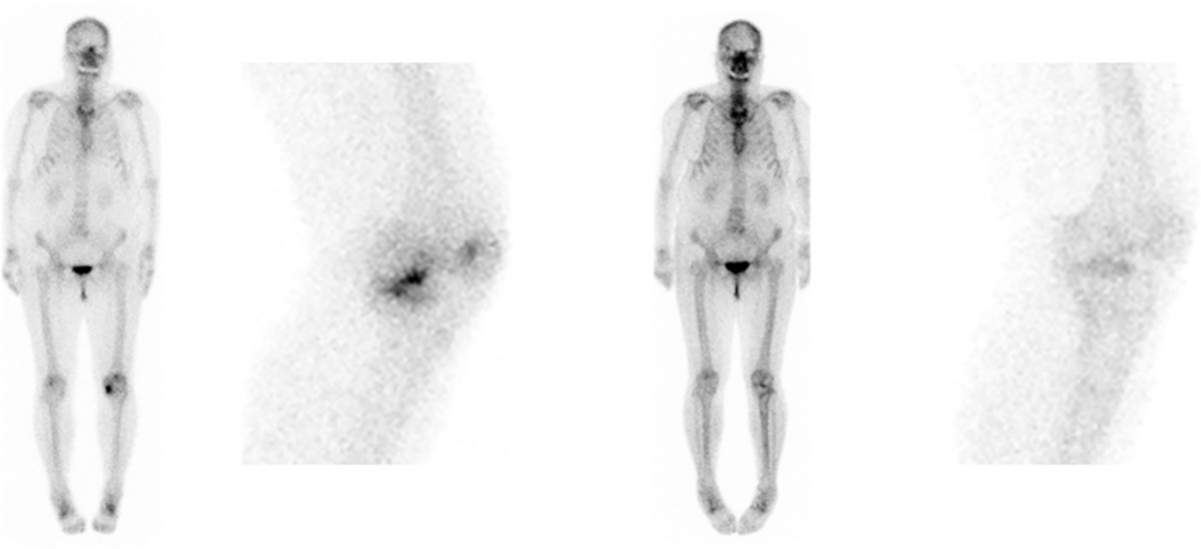
Preoperative (left) BTU of MFC (medial femoral condyle) and MTP (medial tibia plateau) were significantly decreased at 2 years (right) after MOWHTO (medial open-wedge high tibial osteotomy) with adequate correction.

A total of 210 knees were enrolled in the current study and the demographics were summarized in Fig 1 and Table 1. The radiologic parameters, clinical outcomes, ICRS grade and BTU in bone scintigraphy were compared preoperatively and postoperatively (Figs 2, 3 and Table 2). BTU of medial femoral condyle and medial tibial plateau was significantly decreased at 2 years after MOWHTO (P<0.001). Univariable regression analysis showed that BMI, postoperative HKAA and correction angle were associated with postoperative BTU in bone scintigraphy. In multivariable regression analysis, BMI and postoperative HKAA were related to postoperative BTU (Table 3). Lower BMI and larger postoperative HKAA (valgus alignment) showed a significant predictor for the lower postoperative BTU. However, there was no correlation between clinical scores and postoperative BTU. Moreover, there was no significant relationship between cartilage regeneration and postoperative BTU after MOWHTO (Table 4).

**Table 1:** Patient characteristics.

Variables	Data (95% CI)
Patients	210
Sex (male/female)	42/168
Mean age (years)	57.5 ± 6.1 (56.514, 58.299)
BMI (kg/m^2^)	26.1 ± 2.8 (25.708, 26.473)
Correction angle (°)	10.3 ± 2.6 (9.916, 10.647)
Follow-up period (months)	42.7 ± 17.1 (39.395, 43.956)
Interval between the first- and second-look arthroscopy (months)	26.2 ± 5.1 (25.533, 26.945)

Values are given as No. or mean ± SD.

BMI, body mass index; CI, confidence interval.

**Table 2:** Comparison of radiologic, clinical outcomes, arthroscopic findings and BTU in bone scintigraphy preoperatively and postoperatively.

	Preoperative	Postoperative	p-value (95% CI)
*Radiologic outcomes*			
K-L grade (1/2/3/4)	0/110/90/10	3/102/92/13	0.535
FTA (°)	181.6 ± 2.7	171.4 ± 1.5	<0.001 (9.907, 10.618)
PTSA(°)	7.9 ± 3.8	9.7 ± 4.3	<0.001 (-2.348, -1.276)
*Clinical outcomes*			
AKS knee score	58.7 ± 5.2	94.5 ± 2.1	<0.001 (-36.462, -33.118)
AKS function score	61.3 ± 6.5	93.8 ± 5.4	<0.001(-32.132, -29.867)
*ICRS grade*			
(MFC, Grade 1 / Grade 2 / Grade 3 / Grade 4)	0/34/99/77 (first-look)	11/74/81/44 (second-look)	<0.001
(MTP, Grade 1 / Grade 2 / Grade 3 / Grade 4)	0/101/59/50 (first-look)	37/110/33/30 (second-look)	<0.001
*BTU*			
MFC	8.7 ± 5.6	3.4 ± 1.7	< 0.001 (4.699, 5.962)
MTP	7.0 ± 5.2	3.5 ± 1.7	< 0.001 (2.858, 4.062)

Values are given as No. or mean ± SD.

K-L grade, Kellgren-Lawrence grade; FTA, femorotibial angle; AKS, American Knee Society knee score; ICRS, International Cartilage Repair Society; MFC, medial femoral condyle; MTP, medial tibial plateau; BTU, bone tracer uptake; CI, confidence interval.

**Table 3:** Univariable and multivariable postoperative BTU analysis of the medial compartment.

Variable	MFC		MTP	
	P-value			
	Univariable analysis	Multivariable analysis	Univariable analysis	Multivariable analysis
Sex	0.332		0.707	
Age	0.802		0.788	
BMI	0.073	<0.001	0.039	0.001
*Preoperative*				
K-L grade	0.841		0.779	
FTA	0.522		0.110	
PTSA	0.822		0.785	
*Postoperative*				
Correction angle	0.022	0.719	0.061	0.331
FTA	<0.001	<0.001	<0.001	<0.001
PTSA	0.110		0.213	
AKS knee	0.747		0.197	
AKS function	0.932		0.621	

BMI, body mass index; K-L grade, Kellgren-Lawrence grade; FTA, femorotibial angle; PTSA, posterior tibial slope angle; AKS, American Knee Society knee score.

**Table 4:** Comparison of postoperative BTU (bone tracer uptake) and cartilage regeneration.

	Stage A	Stage B	Stage C	p-value
	No regeneration	Partial regeneration	Total regeneration	
MFC				
Total, %	45(21.4)	107(51.0)	58(27.6)	
BTU	3.9 ± 1.8	3.3 ± 1.5	3.2 ± 2.0	0.111
MTP				
Total, %	77(36.7)	91(43.3)	42(20.0.4)	
BTU	3.4 ± 1.4	3.5 ± 1.7	3.6 ± 2.4	0.834

Values are presented as No. (%).

MFC, medial femoral condyle; MTP, medial tibia plateau.

One way ANOVA was used to compare postoperative BTU with stage of articular cartilage regeneration.

## 4. Discussion

The main finding of the current study is that BTU significantly decreased in medial femoral condyle and medial tibial plateau at 2 years after MOWHTO. Moreover, we found that lower BMI and postoperative valgus alignment were significantly related to postoperative BTU decrease of the medial compartment. However, BTU decrease in medial femoral condyle and medial tibial plateau following MOWHTO were not correlated with clinical outcomes or arthroscopic cartilage improvement until the midterm follow-up.

Bone scintigraphy reflects the specific loading pattern of knee with regard to its alignment [11, 12, 30], thus postoperative changes of bone scintigraphy following MOWHTO would be helpful for understanding the mechanism of improvement. However, only few studies reported regarding BTU changes after MOWHTO and it was significantly decreased [15, 30]. Similarly, they analyzed BTU in medial compartment as a whole structure which would be divided into femoral condyle and tibial plateau. On the contrary, current study has a consistent time for assessing postoperative BTU such as postoperative 2 years at the time of scheduled plate removal. Moreover, we assessed BTU of both femoral condyle and tibial plateau in medial compartment independently. Finally, we observed BTU decreased in medial femoral condyle and medial tibial plateau following MOWHTO. Because MOWHTO leads to decompression of medial compartment by shifting the load to the lateral compartment through the valgization of the tibia [30, 31], postoperative BTU might decrease in medial femoral condyle and medial tibial plateau. Moreover, lower BMI and larger postoperative HKAA (valgus alignment) were significant predictors for the BTU decrease of the medial compartment in the current study. We understand that these predictors would be correlated with the amount of joint loading [32, 33]. High BMI produces a chronic overload in the knee joint that is followed by progressive degeneration [34]. Also, HKAA is a key determinant of load distribution of the knee joint that may contribute to the progression of OA [35]. Therefore, BMI and postoperative HKAA would be considered important factors for effective decompression of medial compartment after MOWHTO.

Previous studies have demonstrated that symptom of knee joint was correlated with BTU after MOWHTO [15, 30]. However, those studies had some limitations such as relatively small sample size and inconsistent time in performing postoperative bone scintigraphy. Meanwhile, we conducted our study with enough number of patients and regularly assessed postoperative bone scintigraphy at 2 years after MOWHTO for plate removal. In addition, current study showed that BTU in medial compartment after MOWHTO was not correlated with postoperative clinical outcomes at midterm follow-up. Clinical outcomes after MOWHTO may depend on multiple factors such as sex, race, degree of arthritis, level of physical activity, patient’s individual pain perception and obesity [34, 36]. Because bone scintigraphy mainly reflects the specific loading pattern of knee associated with its alignment [11, 12, 30], we believe that isolate postoperative BTU could not directly reflect clinical outcomes. Moreover, while bone scintigraphy performed at postoperative 2 years, it was compared to the latest clinical outcomes (mean 42.7 months). Therefore, we think postoperative bone scintigraphy cannot predict later outcomes. Meanwhile, there were studies reported that clinical outcome was not correlated with cartilage regeneration [16, 19, 37]. Our finding also indicates that there was no correlation between cartilage regeneration and postoperative BTU. After all, considering our results, the decrease in BTU after MOWHTO seems simply reflection of lateral joint shifting after MOWHTO but does not reflect postoperative clinical or arthroscopic improvement.

Despite the informative results, there are some limitations. First, bone scintigraphy is 2D planar and cannot provide exact anatomical location unlike SPECT/CT. However, we performed bone scintigraphy in same protocol with identical position. Second, the result of study was based on female predominance because prevalence of knee OA in women is much higher in East Asia [38]. Male predominant or Western cohort may show more or less different results. Third, mean follow-up period was not long. However, average 42.7 months follow-up would be enough to reflect the mid-term results of MOWHTO. Long-term follow-up studies might be interesting to confirm gradual changes of BTU and its relevance on the outcomes.

## 5. Conclusion

Lower BMI and postoperative valgus alignment were significant predictor for postoperative BTU decrease of the medial compartment following MOWHTO. However, postoperative changes of BTU did not reflect cartilage regeneration or clinical outcomes until the midterm follow-up. Therefore, the clinical relevance of the results of the bone scintigraphy following MOWHTO remains unclear.

## Supporting information

S1 Dataset (XLSX)
